# Alport's Syndrome in Pregnancy

**DOI:** 10.1155/2013/374020

**Published:** 2013-06-03

**Authors:** Suchita Mehta, Chadi Saifan, Marie Abdellah, Rita Choueiry, Rabih Nasr, Suzanne El-Sayegh

**Affiliations:** ^1^Department of Medicine, Staten Island University Hospital, 475 Seaview Avenue, Staten Island, NY 10305, USA; ^2^Division of Nephrology, Staten Island University Hospital, 475 Seaview Avenue, Staten Island, NY 10305, USA

## Abstract

*Background*. Alport's syndrome is an X-linked hereditary disorder affecting the glomerular basement membrane associated with ocular and hearing defects. In women, the disease is much less severe compared to that in men. However, women with Alport's syndrome can have an accelerated form of their disease during pregnancy with worsening of kidney function and can also develop preeclampsia. There are only four described cases of Alport's syndrome in pregnancy. *Case Presentation*. 20-year-old woman with a history of Alport's syndrome, which during pregnancy worsened resulting in hypertension, proteinuria, and acute kidney injury. Fortunately, there was complete resolution of the proteinuria and kidney injury with delivery, and the patient did not require any renal replacement therapy. *Conclusion*. One of the four reported cases had an accelerated form of the disease during pregnancy with rapid progression of kidney injury and end-stage renal disease. There are no definite guidelines to monitor these patients during pregnancy. Further studies are required to understand the exact pathophysiology of kidney damage that occurs in pregnant women with Alport's syndrome. This may give us some insight into the prognostic predictors, so that we can monitor these women more thoroughly and prevent adverse outcomes.

## 1. Introduction

Guthrie first reported a family with recurrent hematuria, followed by Alport who noted additional features like deafness in this family and early death of the diseased males as compared to the females [1, 2]. Alport's syndrome is a hereditary disease affecting the glomerular basement membrane, also associated with ocular and hearing defects. Hereditary nephritis including Alport's syndrome accounts for 0.4% of ESRD in the adult population of USA [3]. It is usually X-linked, but autosomal dominant and recessive patterns of inheritance have also been described [4, 5]. Being a predominantly X-linked disorder, it mainly manifests in males. In women, the disease is much less severe compared to that in men and seldom causes end-stage renal disease except in a few cases [6]. However, women with Alport's syndrome can have an accelerated form of their disease during pregnancy with worsening of kidney function, proteinuria, hypertension, and hematuria and can develop preeclampsia, eclampsia. There is not sufficient literature on the course and prognosis of Alport's syndrome in pregnant women. There are only four described cases of Alport's syndrome in pregnancy. Some of the reported cases had an accelerated form of the disease during pregnancy with rapid progression of kidney injury, while others had a good outcome. The exact pathophysiology of this progression in pregnancy is not known. Also, there are no clear prognostic predictors as to which of these pregnant women with Alport's syndrome will have further worsening of their disease. Here, we report a case of a woman with a history of Alport's syndrome, which during pregnancy worsened resulting in hypertension, proteinuria, and acute kidney injury. However, there was significant decrease in the proteinuria and kidney injury with delivery of the fetus.

## 2. Case 

A 20-year-old woman, gravid 1 Para 0, with a known past medical history of Alport's Syndrome diagnosed at 13 years of age, presented to the obstetrics clinic for regular prenatal check. She was diagnosed to have Alport's syndrome by means of genetic testing and kidney biopsy at 13 years of age; this was done as she was found to have proteinuria on routine urine analysis, and her mother and brother were known to have Alport's syndrome with both of them being on dialysis for end-stage renal disease. Prior to pregnancy, she did not have a regular followup with her doctor; however, she was noted to be normotensive with a blood pressure of 116/78 mmHg in 2006 when she underwent renal biopsy, and the urine protein excretion was 1.04 grams/day. At 14-week gestation, she was found to have urine protein of 1.4 grams/day and blood pressure of 110/70 mmHg. Her pregnancy course was uncomplicated till 29-week gestation, her blood pressure was within normal limits on her prenatal visits, and ultrasound of the abdomen had also been normal. When she presented to the clinic at 29-week gestation, she was asymptomatic but was found to be hypertensive with a blood pressure of 162/111 mmHg and also had 1+ bilateral lower extremity edema. She was admitted for further management with concern to rule out preeclampsia. Laboratory workup revealed a rise in serum creatinine to 1.53 mg/dL from a baseline serum creatinine 0.7 mg/dL, normal electrolytes, and a normal complete blood count. Routine urine analysis showed >300 mg/dL proteinuria on dipstick, and 24-hour urine showed nephritic range proteinuria of 15 g. She was diagnosed with preeclampsia and acute renal failure. The cause of this acute renal insufficiency was thought to be due to preeclampsia or worsening Alport's syndrome causing uncontrolled high blood pressure and acute kidney injury. She was treated with intravenous labetalol to control the blood pressure, magnesium sulfate for preeclampsia. Induction of labor was attempted but failed, leading to a Caesarean section. She gave birth to a healthy neonate without any further complications. After the delivery, proteinuria decreased to 750 mg/day and the serum creatinine decreased to 1.1 mg/dL the next day. She also became normotensive thereafter with a blood pressure of 130/78 mmHg. Her acute renal failure thus resolved, without the need for any renal replacement therapy.

## 3. Discussion

Alport's syndrome is a hereditary disease affecting the glomerular basement membrane due to a defect in alpha chains of type IV collagen. COL4A5 gene, located on X chromosome encoding *α*-5 chains, is one of the six genes implicated in Alport's syndrome [7]. Type IV collagen is present in many parts of the body besides the glomerular basement membranes including the skin, eyes, and cochlea. Skin biopsy showing absence of *α*-5 staining is one of the methods to detect X-linked Alport's syndrome [8]. It can present at any age but most frequently presents at a young age [9]. Light microscopy of the renal biopsy in Alport's syndrome is nonspecific, and the histological diagnosis mainly rests on electron microscopy which typically shows lamellation, thinning, and thickening of the glomerular basement membrane [10]. The clinical manifestations include hematuria usually microscopic, occasionally abdominal pain, or flank pain. Ocular abnormalities occur in about 15 to 30% of patients, and the most common abnormality seen is anterior lenticonus [11]. Hearing abnormality due to cochlear lesions cause a high frequency sensorineural deafness in 30 to 50% of patients [12]. Even though hearing abnormalities are always accompanied by renal disease, the degree of sensorineural deafness is not related to degree of renal damage [13]. Hypertension is usually present in advanced cases. End-stage renal disease (ESRD) is seen in males between 16 to 35 years of age [14]. ESRD is not commonly seen in females as most of cases of Alport's syndrome are X-linked. However, in pregnancy, the disease can progress and become more severe, not only leading to worsening renal failure but also preeclampsia. The exact pathophysiology and molecular mechanism for this worsening is not known. There are speculations that due to the presence of the *α* chains of type IV collagen in the placental membranes, there is damage to the placenta causing more oxidative stress resulting in preeclampsia and thereby leading to hypertension and renal failure, but this has not been proven [15]. There are only two cases of Alport's syndrome in pregnancy that have been reported in English. In 2007, Matsuo et al. reported their patient who developed renal failure, proteinuria that did not resolve after delivery, and the patient went on to become dialysis dependent [15]. The case reported by Matsubara et al. in 2009 [16] had a favorable course as the pregnancy lasted full term, and there was minimal renal damage. Here, we report a patient with Alport's syndrome who was asymptomatic prior to pregnancy without any proteinuria. She started developing proteinuria during her third trimester, uncontrolled hypertension, acute renal failure, and preeclampsia, requiring urgent delivery at 29 weeks. Afterpartum there was no significant decrease in proteinuria, and the creatinine came back to baseline with resolution of acute renal failure.

Looking at all the cases described previously, it is not clear which of the pregnant patients with Alport's syndrome will progress to develop renal failure and preeclampsia. The least that can be done for these patients is urine analysis to check for proteinuria, blood pressure monitoring, and basic metabolic panel, but further studies would be needed to determine how often they should be performed in pregnant patients with Alport's syndrome. More importantly, in the event of any abnormality like increased proteinuria, it is essential to know how aggressive one should be in the management of the pregnancy and the renal disease in this patient population; whether one can manage patients conservatively and monitor renal function or resort to more drastic measures. There are no evidence-based guidelines for monitoring these pregnant women with Alport's syndrome to prevent complications. Further studies are required to understand the molecular basis of Alport's syndrome in pregnancy, so that we can predict the course for these women during pregnancy and develop better strategies to prevent these adverse outcomes. Studies with a larger sample size of pregnant women with Alport's syndrome are required to analyze their pregnancy course and outcome in order to develop better evidence-based monitoring guidelines in such patients.

## Abbreviations

ESRD: End-Stage renal disease
USA: United States of America
mg: Milligrams
dL: Deciliters.
